# Uncomplicated Urinary Tract Infections in Women in a Sao Paulo Quaternary Care Hospital: Bacterial Spectrum and Susceptibility Patterns

**DOI:** 10.3390/antibiotics3010098

**Published:** 2014-03-19

**Authors:** Marcelo Hisano, Homero Bruschini, Antonio Carlos Nicodemo, Miguel Srougi

**Affiliations:** 1 Division of Urology, Hospital das Clinicas-SP, University of Sao Paulo, 255 Doutor Enéas Carvalho de Aguiar Ave., 7th floor, São Paulo 05403-000, Brazil; E-Mails: marcelohisano@hotmail.com (M.H.); srougi@uol.com.br (M.S.); 2 Infectious Disease Department, Hospital das Clinicas-SP, University of Sao Paulo, 255 Doutor Enéas Carvalho de Aguiar Ave., 4th floor, São Paulo 05403-000, Brazil; E-Mail: ac_nicodemo@uol.com.br

**Keywords:** urinary tract infection, female, cystitis, pyelonephritis

## Abstract

Uncomplicated urinary tract infections (UTI) in women are very common. Regular analysis of bacterial flora is important to formulate updated guidelines. The objective of this study is to determine and compare the microbiology of UTIs and their susceptibility patterns in a quaternary care hospital. In a seven-year review, the urine culture results of 480 female patients with uncomplicated UTIs were analyzed. Patients were divided into three groups according to their diagnosis and treatment characteristics: Group 1, cystitis at outpatient basis; group 2, cystitis at the Emergency Unit; and group 3, pyelonephritis. Group 1 included older patients, with a higher incidence of concomitant diabetes mellitus and recurrent UTIs. *E. coli* was the most common pathogen, responsible for 75.1% of cases, mainly for pyelonephritis (87.3%). Of the oral antimicrobials tested for cystitis, amoxicillin/clavulanate and nitrofurantoin had the highest susceptibility profiles (84.4% and 87.3%, respectively). For *E. coli* only, their susceptibility profiles were as high as 90.8% and 97.4%, respectively. For pyelonephritis treatment, fluoroquinoles had a susceptibility profile <90%, while ceftriaxone and gentamicin had susceptibility >90%. Uncomplicated UTI treatment is becoming more challenging because the susceptibility profiles of oral antimicrobials are increasingly resistant. In our environment, cystitis can still be managed with nitrofurantoin. Uncomplicated pyelonephritis should be managed with ceftriaxone or gentamicin.

## 1. Introduction

Urinary tract infections (UTI) in women are very common. The annual incidence was 10.8% in 2000, and 60% of women will have at least one episode during their lifetime [1]. Additionally, after a first episode of urinary tract infection, 44% of the women will experience another episode within the following year [2].

UTI can be classified as uncomplicated or complicated. Uncomplicated UTI is defined as a UTI that occurs in a woman who has no structural or functional abnormalities, is not pregnant, and has not been instrumented [3]. Anatomically, uncomplicated UTI can be located in the lower urinary tract (cystitis) or in the upper urinary tract (pyelonephritis) [4,5,6].

Usually, uncomplicated cystitis can be managed on an outpatient basis in a primary care health system, while uncomplicated pyelonephritis is usually managed in a hospital setting, followed by outpatient care. At our Institution, a quaternary care hospital located in a developing country, uncomplicated cystitis can be managed either on an office outpatient basis or at the Urological Emergency Unit, according to the needs of the Public Health Care System.

As uncomplicated lower UTIs are usually treated empirically, knowledge of the contemporary flora and pattern of susceptibility is essential and mandatory. While UTI surveillance studies from Europe and the USA are available [4,7,8,9], equivalent studies from developing countries are sparse [10]. These data are important to formulate guidelines, particularly for such a prevalent disease, as they have the potential to change clinical practice [11]. The aim of this study was to determine the bacterial flora of uncomplicated cystitis and pyelonephritis and compare the susceptibility patterns to identify potential treatment characteristics.

## 2. Experimental

Between January, 2007, and December, 2012, we retrospectively reviewed the clinical records of 160 female patients attended at the Urological Office on an outpatient basis and 2,949 female patients treated at the Urological Emergency Unit. Of these patients, we identified and selected for analysis those with a clinical diagnosis of uncomplicated cystitis and pyelonephritis. This review had the approval of the Institutional Ethical Committee.

Uncomplicated cystitis was defined as involving clinical symptoms of dysuria, frequency, urgency, and suprapubic pain, with or without hematuria. Uncomplicated pyelonephritis was defined as clinical symptoms of flank pain, associated with nausea, vomiting, and fever (>37.8 °C), with or without symptoms of cystitis. All patients with these symptoms had a midstream clean catch urine culture. Urine cultures were analyzed, and a colony count of ≥10^3^ cfu/mL was considered positive in cases of cystitis; in the case of pyelonephritis, the threshold was ≥10^4^ cfu/mL. Identification of isolates and susceptibility tests were performed on VITEK^®^ 1 and 2 automated systems (bioMérieux^®^, Marcy L'Etoile, France). The minimal inhibitory concentrations (MICs) of antimicrobials were determined, and strains were considered susceptible, intermediately susceptible, or resistant according to the breakpoints determined by the Clinical and Laboratory Standards Institute—CLSI [12] at the time of the analysis. The following antimicrobials were tested: amikacin, amoxicillin/clavulanate (A/C), ampicillin, cefepime, cefotaxime, cefoxitin, ceftazidime, ceftriaxone, cephalothin, ciprofloxacin, gentamicin, levofloxacin, nalidixic acid, nitrofurantoin, norfloxacin, piperacillin/tazobactam (Pip/Taz), and sulfamethoxazole/trimethoprim (SMT). For some bacteria, such as *Staphylococcus saprophyticus*, *Streptococcus agalactiae*, coagulase-negative *Staphylococcus*, *Corynebacterium* spp., and yeast species, susceptibility profiles were not performed because there are no established breakpoints, according to CLSI guidelines [12].

During the review process of the record files, we were able to identify and analyze some of these three clinical characteristics: age (<50 years-old and ≥50 years-old), history of recurrent UTI, and presence of diabetes mellitus (DM). Recurrent UTI was defined as the presence of two or more UTI episodes during a period of six months.

We excluded patients based on the following criteria: being under 14 years-old, having kidney stones larger than 5 mm or any ureteral or bladder stone, having a UTI less than two months prior to the current episode, having recent urinary catheterization, being within the convalescence period of urological surgery; and having a double-J stent, urinary diversion, kidney transplantation, or any congenital urological abnormality without correction. We also excluded patients for whom urine cultures were negative or not available, despite clinical symptoms of uncomplicated UTI.

Three groups were created: group 1—patients treated at the Urological Office as outpatients with uncomplicated cystitis; group 2—patients treated at the Urological Emergency Unit with uncomplicated cystitis; and group 3—patients treated at the Urological Emergency Unit with uncomplicated pyelonephritis. We then analyzed and compared the microbiological findings of the patients’ urine samples.

Statistical analyses of the nonparametric results were performed by a chi-squared test or likelihood ratio test. When the samples were insufficient for chi-squared analysis, the Fisher exact test was performed. A *p* value of <0.05 was considered statistically significant.

## 3. Results

Of the 160 patients attending the Urological Office as outpatients, 103 were included in this study with uncomplicated cystitis. Of the 2,949 patients treated at the Urological Emergency Unit, 276 were included in this study with uncomplicated cystitis and 101 with uncomplicated pyelonephritis. Seven patients in group 1, five in group 2, and one in group 3 had two episodes of UTI. The second positive samples from each of those patients were included in the analysis, giving a total of 110 urine samples in group 1, 281 in group 2, and 102 in group 3. The 480 patients included in this study provided 493 urine samples.

The mean ages (and ranges) of patients in groups 1, 2, and 3 were: 55 (18–96) years, 43.2 (15–92) years, and 36 (14–79) years, respectively. The three groups were not equivalent regarding age, history of recurrent UTI or diabetes mellitus. Group 1 had older patients, increased occurrence of recurrent UTI, and greater incidence of diabetes mellitus (Table 1).

**Table 1:** Demographic data of the three groups and of the total cohort.

Clinical data	Groups				
	1	2	3	Total	*p*
	N (%)	N (%)	N (%)	N (%)	
**Patients (N)**	103	276	101	480	n.a.
**Age <50 years** *	38 (36.8)	172 (62.3)	81 (81.0)	291 (60.6)	**<0.001**
**Recurrent UTI** *	83 (76.1)	82 (36.0)	22 (26.5)	187 (44.5)	**<0.001**
**DM** *	25 (22.7)	18 (8.5)	2 (2.2)	45 (10.9)	**<0.001**

* Some patients with missing data; n.a.—not applicable; DM—diabetes mellitus.

The microbiology of groups 1, 2, and 3 is summarized in Table 2. In all groups, monomicrobial infection was the most common finding, varying from 92.5% to 95.1%. *Escherichia coli* (*E. coli*) monoinfection was the most common microorganism in monoinfection UTI, varying from 71.2% to 87.6%. Urinary infection caused by two agents (mixed infection) was identified in 7.3% of samples in group 1, 7.5% in group 2, and 4.9% in group 3.

**Table 2:** Mono and mixed infections in the three groups of patients and in the total cohort.

Urine culture	Group 1	Group 2	Group 3	Total
Urine Samples (%)	110 (22.3)	281 (57.0)	102 (20.7)	493 (100.0)
Monoinfection (%)	102 (92.7)	260 (92.5)	97 (95.1)	459 (93.1)
*E. coli* (% monoinfection)	78 (76.5)	185 (71.2)	85 (87.6)	348 (75.8)
Non-*E. coli* (% monoinfection)	24 (23.5)	75 (28.8)	12 (12.4)	111 (24.2)
Mixed Infection (%)	8 (7.3)	21 (7.5)	5 (4.9)	34 (6.9)
*E. coli* * (%)	5 (62.5)	13 (61.9)	4 (80.0)	22 (64.7)
Non-*E. coli* (% mixed infection)	3 (37.5)	8 (38.1)	1 (20.0)	12 (35.3)

* *E. coli* as one agent.

When we analyzed all mono and mixed infection bacterial species together (Table 3), *E. coli* was still the most frequent pathogen, mainly for group 3 when compared to the other groups (*p* = 0.004). There was also a significantly higher frequency of *Staphylococcus saprophyticus* in group 2 (*p* = 0.004).

**Table 3:** Bacterial spectrum in the three groups of patients and in the entire cohort.

Microorganism	Groups				
	1	2	3	Total	*p*
	N (%)	N (%)	N (%)	N (%)	
*Escherichia coli*	83 (75.5)	198 (70.5)	89 (87.3)	370 (75.1)	**0.004**
*Klebsiella pneumoniae*	7 (6.4)	6 (2.1)	6 (5.9)	19 (3.9)	0.074
*Enterococcus faecalis*	11 (10.0)	18 (6.4)	3 (2.9)	32 (6.5)	0.113
*Proteus mirabilis*	3 (2.7)	14 (5.0)	3 (2.9)	20 (4.1)	0.472
*Staphylococcus saprophyticus*	3 (2.7)	28 (10.0)	2 (2.0)	33 (6.7)	**0.004**
*Streptococcus agalactiae*	2 (1.8)	11 (3.9)	0 (0.0)	13 (3.3)	0.367
**Other**	8 (7.3)	26 (9.3)	4 (3.9)	38 (7.7)	0.220
**Total**	117 (100)	301 (100)	107 (100)	493 (100)	n.a

n.a: not applicable.

We compared the susceptibility profile of *E. coli* to all other bacteria in groups 1, 2, and 3 (Table 4). In group 1, there was a significant difference in susceptibility for A/C, cefoxitin, nitrofurantoin and SMT. For A/C, the difference was mostly due to a decrease in the resistance profile of *E. coli* compared to other bacteria. Cefoxitin and nitrofurantoin had higher susceptibility for *E. coli* than for the other bacteria (96.1% × 62.5%, respectively, for cefoxitin, *p* = 0.001; 97.4% × 30.8%, respectively, for nitrofurantoin, *p* < 0.001). For SMT, the susceptibility of *E. coli* was lower than the other bacteria (56.2% and 92.3%, respectively; *p* = 0.013). In group 2, we found a significant difference in the susceptibility profile between *E. coli* and other bacteria for A/C (90.8% × 78.3%, respectively; *p* = 0.031), cefoxitin (96.5% × 70.6%, respectively; *p* = 0.002), cephalothin (66.2% × 73.1%, respectively; *p* = 0.003), nitrofurantoin (94.5% × 23.8%, respectively; *p* < 0.001) and SMT (64.1% × 85.2%, respectively; *p* = 0.030). In group 3, when comparing the susceptibility profile of *E. coli* and all other bacteria, there were significant differences in the susceptibility profiles for cefoxitin (96.4% × 66.7%, respectively; *p* = 0.043) and nitrofurantoin (95.2% × 42.9%, respectively; *p* < 0.001). Between groups 1, 2, and 3, when analyzing *E. coli*, there was a susceptibility difference for amikacin (100% × 100% × 92.9%, respectively; *p* < 0.001); when analyzing other bacteria, there was a susceptibility difference for ciprofloxacin (95.2% × 82.5% × 60%, respectively; *p* = 0.049).

## 4. Discussion

*E. coli* is the predominant uropathogen in uncomplicated UTI, involved in 75.1% of cases. For pyelonephritis, *E. coli* is even more common than the other bacteria, with a frequency of 87.3%; some studies have shown that *E. coli* is the responsible pathogen in 80 to 90% of these cases [13,14]. Although the genetic and behavioral risk factors for cystitis and pyelonephritis are similar, the predominance of *E. coli* causing pyelonephritis can be attributed to its intrinsic pathogenicity and virulence [15].

In general, when analyzing the susceptibility profile, there is no oral treatment option with susceptibility higher than 90%, although some authors considered a threshold of 80% for inclusion in the Clinical Guidelines [4]. A/C and nitrofurantoin were closer to this level, and they are more effective, especially for *E. coli*. For pyelonephritis treatment, ceftriaxone had a susceptibility of at least 94%; thus, it can be selected to initiate therapy in place of fluoroquinolones. 

Our quaternary care hospital has its particularities. The Urological Emergency Unit treats all types of urological emergencies, from acute simple cases to complex urogenital trauma. Because it is not an exclusive referenced emergency, the UTI cases assisted there usually reflect community-acquired infections. The Urological Office takes care of more patients with comorbidities, representing more complex cases. A similar type of UTI classification has been published by Laupland *et al.* [16], analyzing ambulatory, hospital, and nursing home UTIs. They found *E. coli* frequencies of 74.2%, 65.5%, and 46.6% in each of these locations, respectively, as well as a difference in susceptibility profiles between them.

**Table 4:** Susceptibility/resistance profiles of *E. coli* and other pathogens for 17 antibiotics tested in the three groups of patients and in the entire cohort.

Antibiotic			Group 1				Group 2				Group 3				Total			*p*
			Susceptible		Resistant		Susceptible		Resistant		Susceptible		Resistant		Susceptible		Resistant	
			N (%)		N (%)		N (%)		N (%)		N (%)		N (%)		N (%)		N (%)	
**AMIKACIN**	*E. coli*		82 (100.0)		0 (0.0)		183 (100.0)		0 (0.0)		79 (92.9)		6 (7.1)		344 (98.3)		6 (1.7)	**<0.001**
	Other		13 (100.0)		0 (0.0)		25 (96.2)		1 (3.8)		7 (100.0)		0 (0.0)		45 (97.8)		1 (2.2)	0.56
	*p*		>0.999				0.124				>0.999				0.582			
**A/C**	*E. coli*		54 (73.0)		2 (2.7)		168 (90.8)		5 (2.7)		77 (91.7)		4 (4.8)		299 (96.5)		11 (3.5)	0.725
	Other		9 (69.2)		3 (23.1)		18 (78.3)		4 (17.4)		5 (71.4)		2 (28.6)		32 (78)		9 (22)	0.81
	*p*		**0.028**				**0.031**				0.132				**<0.001**			
**AMPICILLIN**	*E. coli*		35 (42.2)		48 (57.8)		94 (48.0)		100 (51.0)		32 (42.7)		41 (54.7)		161 (46.0)		189 (54.0)	0.577
	Other		9 (40.9)		13 (59.1)		22 (59.5)		15 (40.5)		5 (45.5)		6 (54.5)		36 (51.4)		34 (48.6)	0.352
	*p*		0.915				0.331				0.754				0.406			
**CEFEPIME**	*E. coli*		77 (96.2)		3 (3.8)		173 (96.6)		6 (3.4)		79 (96.3)		3 (3.7)		329 (96.5)		12 (3.5)	0.984
	Other		13 (100.0)		0 (0.0)		25 (100.0)		0 (0.0)		6 (85.7)		1 (14.3)		44 (97.8)		1 (2.2)	0.146
	*p*		>0.999				>0.999				0.284				>0.999			
**CEFOTAXIME**	*E. coli*		49 (94.2)		3 (5.8)		132 (95.7)		6 (4.3)		65 (97.0)		2 (3.0)		246 (95.7)		11 (4.3)	0.755
	Other		8 (100.0)		0 (0.0)		15 (100.0)		0 (0.0)		5 (83.3)		1 (16.7)		28 (96.6)		1 (3.4)	0.193
	*p*		>0.999				>0.999				0.230				>0.999			
**CEFOXITIN**	*E. coli*		49 (96.1)		0 (0.0)		138 (96.5)		2 (1.4)		54 (96.4)		2 (3.6)		241 (98.4)		4 (1.6)	0.268
	Other		5 (62.5)		3 (37.5)		12 (70.6)		4 (23.5)		4 (66.7)		2 (33.3)		21 (70.0)		9 (30.0)	0.805
	*p*		**0.001**				**0.002**				**0.043**				**<0.001**			
**CEFTAZIDIME**	*E. coli*		49 (94.2)		3 (5.8)		137 (95.8)		6 (4.2)		53 (96.4)		3 (5.4)		239 (95.2)		12 (4.8)	0.880
	Other		8 (100.0)		0 (0.0)		17 (100.0)		0 (0.0)		5 (83.3)		1 (16.7)		30 (96.8)		1 (3.2)	0.18
	*p*		>0.999				>0.999				0.342				>0.999			
**CEFTRIAXONE**	*E. coli*		30 (100.0)		0 (0.0)		58 (100.0)		0 (0.0)		31 (100.0)		0 (0.0)		119 (100.0)		0 (0.0)	>0.999
	Other		7 (100.00)		0 (0.0)		11 (100.0)		0 (0.0)		2 (100.0)		0 (0.0)		20 (100.0)		0 (0.0)	>0.999
	*p*		>0.999				>0.999				>0.999				>0.999			
**CEPHALOTIN**	*E. coli*		56 (67.5)		18 (21.7)		129 (66.2)		28 (14.4)		59 (66.3)		13 (14.6)		244 (80.5)		59 (19.5)	0.479
	Other		9 (60.0)		5 (33.3)		19 (73.1)		7 (26.9)		6 (75.0)		2 (25.0)		34 (70.8)		14 (29.2)	0.813
	*p*		0.601				**0.003**				0.179				0.124			
**CIPROFLOXACIN**	*E. coli*		63 (75.9)		19 (22.9)		164 (83.2)		32 (16.2)		75 (84.3)		14 (15.7)		302 (82.3)		65 (17.7)	0.337
	Other		20 (95.2)		1 (4.8)		33 (82.5)		5 (12.5)		6 (60.0)		4 (40.0)		59 (85.5)		10 (14.5)	**0.049**
	*p*		0.081				0.136				0.080				0.516			
**GENTAMICIN**	*E. coli*		77 (92.8)		5 (6.0)		184 (94.4)		10 (5.1)		87 (97.8)		1 (1.1)		348 (95.6)		16 (4.4)	0.143
	Other		13 (86.7)		2 (13.3)		31 (96.9)		0 (0.0)		10 (100.0)		0 (0.0)		54 (96.4)		2 (3.6)	0.065
	*p*		0.555				0.105				0.806				>0.999			
**LEVOFLOXACIN**	*E. coli*		58 (78.4)		15 (20.3)		156 (84.3)		27 (14.6)		72 (85.7)		11 (13.1)		286 (84.4)		53 (15.6)	0.406
	Other		13 (92.9)		1 (7.1)		21 (84.0)		3 (12.0)		5 (62.5)		3 (37.5)		39 (84.8)		7 (15.2)	0.186
	*p*		0.362				0.590				0.246				0.942			
**NALIDIXIC ACID**	*E. coli*		52 (70.3)		22 (29.7)		149 (81.0)		35 (19.0)		66 (80.5)		16 (19.5)		267 (78.5)		73 (21.5)	0.147
	Other		12 (92.3)		1 (7.7)		19 (82.6)		4 (17.4)		5 (71.4)		2 (28.6)		36 (83.7)		7 (16.3)	0.466
	*p*		0.170				>0.999				0.626				0.43			
**NITROFURANTOIN**	*E. coli*		74 (97.4)		0 (0.0)		172 (94.5)		3 (1.6)		80 (95.2)		1 (1.2)		326 (98.8)		4 (1.2)	0.344
	Other		4 (30.8)		5 (38.5)		5 (23.8)		13 (61.9)		3 (42.9)		4 (57.1)		12 (35.3)		22 (64.7)	0.621
	*p*		**<0.001**				**<0.001**				**<0.001**				**<0.001**			
**NORFLOXACIN**	*E. coli*		26 (83.9)		5 (16.1)		54 (85.7)		8 (12.7)		25 (83.3)		5 (16.7)		105 (85.4)		18 (14.6)	0.859
	Other		10 (83.3)		2 (16.7)		17 (77.3)		3 (13.6)		3 (75.0)		1 (25.0)		30 (83.3)		6 (16.7)	0.896
	*p*		>0.999				0.311				0.559				0.764			
**PIP/TAZ**	*E. coli*		47 (94.0)		8 (100.0)		133 (98.5)		0 (0.0)		52 (100.0)		0 (0.0)		232 (98.7)		3 (1.3)	0.009
	Other		3 (6.0)		0 (0.0)		16 (94.1)		1 (5.9)		6 (100.0)		0 (0.0)		30 (96.8)		1 (3.2)	0.541
	*p*		>0.999				0.087				>0.999				0.393			
**SMT**	*E. coli*		45 (56.2)		35 (43.8)		118 (64.1)		66 (35.9)		55 (64.7)		30 (35.3)		218 (62.5)		131 (37.5)	0.424
	Other		12 (92.3)		1 (7.7)		23 (85.2)		4 (14.8)		7 (100.0)		0 (0.0)		42 (89.4)		5 (10.6)	0.341
	*p*		**0.013**				**0.030**				0.091				**<0.001**			

Note: Intermediate susceptible corresponds to 100% (susceptible + resistant). A/C–amoxicillin/clavulanic acid; Pip/Taz–piperacillin/tazobactam; SMT–sulfamethoxazole/trimethoprim.

In 2008, a multi-center analysis of the microbiology of uncomplicated cystitis (ARESC) [8] showed an *E. coli* frequency of 76.7%, close to that found in the present study. The overall susceptibilities to ampicillin, A/C, ciprofloxacin, nalidixic acid, and nitrofurantoin were 45.1%, 82.1%, 91.8%, 81.4%, and 95.2%, respectively. In the same study, the susceptibility profiles of Brazilian samples for ampicillin, A/C, ciprofloxacin, nalidixic acid, and nitrofurantoin were 33.8%, 78.7%, 89.0%, 74.7%, and 84.1%, respectively. In our study considering both groups 1 and 2 together, we found susceptibilities of 47.3%, 84.4%, 82.1%, 78.9%, and 87.3%, respectively, for the same antimicrobials. Our results showed susceptibility profiles for these antimicrobials similar to those found for Brazilian samples, although the period and place of analysis were different (the ARESC study was conducted between September, 2003 and June, 2006 and involved four Brazilians centres).

When we compared *E. coli* to the other bacteria (mainly *Klebsiella pneumonia*, *Enterococcus faecalis* and *Proteus mirabilis*) in all groups, the susceptibility profiles differed, mainly for A/C, cefoxitin, and nitrofurantoin. These antimicrobials had higher susceptibility results when the UTI agent was *E. coli*. In another study of the ARESC group [17], the susceptibility profiles of Brazilian *E. coli* samples for ampicillin, A/C, ciprofloxacin, nalidixic acid, nitrofurantoin, and SMT were 37.7%, 79.8%, 89.2%, 75.4%, 94.3%, and 54.5%, respectively. For the same antimicrobials, our study found susceptibilities of 46.0%, 96.5%, 82.3%, 78.5%, 98.8%, and 62.5%, respectively. A similar result was found by Linhares *et al.* [18] for nitrofurantoin and pivmecillinam, with the same pattern of high susceptibility for *E. coli* but low for non-*E. coli*, although they analyzed male and female patients. Nitrofurantoin is known to have no activity against *Proteus* spp. and *Pseudomonas aeruginosa* [19]; this information is in line with our results because *P. mirabilis* was analyzed as a non-*E. coli* agent. Interestingly, although SMT had a low susceptibility profile for *E. coli* (56.2%, 64.1%, and 64.7% for groups 1, 2, and 3, respectively), it had a higher susceptibility profile to non-*E. coli* agents, reaching 92.3%, 85.2%, and 100% susceptibility in groups 1, 2, and 3, respectively (*p* < 0.05 for groups 1 and 2 only). These findings may indicate that SMT cannot be considered for *E. coli* treatment but can be used for non-*E. coli* bacteria, although others studies did not find similar results [17,18].

Our study has some limitations. The retrospective nature of this analysis has an intrinsic bias. As we retrospectively analyzed six years of uncomplicated UTI, some changes in MIC occurred during these years according to CLSI. We are aware of this fact, but as we considered final outcomes of the test (Susceptible, Intermediately Susceptible, and Resistant) to treat the patients at that moment successfully, we considered it clinically relevant; other groups had already utilized this classification for the results of susceptibility tests [10,16,18]. Another limitation is the fact that susceptibility profiles were not performed for some bacteria.

The three groups were not statistically similar; group 1 had older patients with higher incidences of diabetes mellitus and recurrent UTI than groups 2 and 3. This difference adds another source of bias to this study, however, as already mentioned, this difference was due to our local health care organization. Additionally, confirming our perception, this group division resembled a comparison between uncomplicated UTIs from a quaternary care hospital (group 1) and community-acquired uncomplicated UTIs (groups 2 and 3).

The 2013 European Guideline on urological infections [5] outlines the antimicrobials of choice for the treatment of uncomplicated cystitis: fosfomycin trometamol, pivmecillinam and nitrofurantoin. Our study did not test fosfomycin trometamol or pivmecillinam; the latter is not available in our country. Fosfomycin trometamol is not routinely tested at our hospital because, at the time of urine culture analysis, there were no established MIC breakpoints for some bacteria and antimicrobials according to CLSI guidelines [12]. Additionally, due to the retrospective nature of this study, we were unable to perform retests. Nitrofurantoin had a general susceptibility profile close to 90%, but for *E. coli*, it was at least 94.5%. In our environment, A/C and ciprofloxacin had susceptibilities of 84.4% and 82.1%, respectively; both were at the lower limit for inclusion as first options for cystitis treatment. Although some European countries reported similar susceptibility profiles, these antibiotics are recommended by their national guidelines [20].

For uncomplicated pyelonephritis, the Infectious Disease Society of America Guidelines [4] and the 2013 European Guideline [5] recommend fluoroquinolones as alternative therapeutic agents. Our study showed a susceptibility profile lower than 90% for these antimicrobials, making them unsuitable for our environment because the susceptibility threshold to be considered as a first line therapy should be higher than 90% [4,6]. The alternatives for empirical antimicrobial therapy are ceftriaxone, with a susceptibility of 100%, or gentamicin, with a susceptibility of 98.0%. Although nitrofurantoin had an acceptable susceptibility profile for pyelonephritis treatment, mainly for *E. coli*, due to its rapid renal excretion and insufficient therapeutic blood level, it is not indicated to treat pyelonephritis [21].

## 5. Conclusions

Uncomplicated UTI treatment is becoming more difficult to manage because the susceptibility profiles of current oral antimicrobials are becoming more resistant. For simple cystitis, nitrofurantoin is at the lower limit of acceptable susceptibility to continue to be an antimicrobial of choice, but it is still very active for *E. coli*, as well as A/C. For pyelonephritis, fluoroquinolones are not suitable as a first line therapy and should be replaced by ceftriaxone or gentamicin. Judicious use of antimicrobials, adhesion to guidelines, and new drug alternatives should be considered as strategies to avoid increasing resistance.
